# STAT activation in regulatory CD4^+^ T cells of patients with primary sclerosing cholangitis

**DOI:** 10.1002/iid3.1248

**Published:** 2024-04-12

**Authors:** Leona Dold, Sandra Kalthoff, Leonie Frank, Taotao Zhou, Pia Esser, Philipp Lutz, Christian P. Strassburg, Ulrich Spengler, Bettina Langhans

**Affiliations:** ^1^ Department of Internal Medicine I University Hospital of Bonn Bonn Germany; ^2^ German Center for Infection Research (DZIF) Partner Site Cologne‐Bonn Bonn Germany

**Keywords:** ectonucleotidase CD39, interleukin‐6, primary sclerosing cholangitis, regulatory CD4^+^ T cells, STAT proteins

## Abstract

**Introduction:**

Regulatory CD4^+^ T cells (Tregs) are pivotal for inhibition of autoimmunity. Primary sclerosing cholangitis (PSC) is an autoimmune cholestatic liver disease of unknown etiology where contribution of Tregs is still unclear. Activation of the JAK‐STAT pathway critically modifies functions of Tregs. In PSC, we studied activation of STAT proteins and Treg functions in response to cytokines.

**Methods:**

In 51 patients with PSC, 10 disease controls (chronic replicative hepatitis C), and 36 healthy controls we analyzed frequencies of Foxp3^+^CD25^+^CD127^low^CD4^+^ Tregs, their expression of ectonucleotidase CD39, and cytokine‐induced phosphorylation of STAT1, 3, 5, and 6 using phospho‐flow cytometry. In parallel, we measured cytokines IFN‐gamma, interleukin (IL)‐6, IL‐2, and IL‐4 in serum via bead‐based immunoassays.

**Results:**

In patients with PSC, *ex vivo* frequencies of peripheral Tregs and their expression of CD39 were significantly reduced (*p* < .05 each). Furthermore, serum levels of IFN‐gamma, IL‐6, IL‐2, and IL‐4 were markedly higher in PSC (*p* < .05 each). Unlike activation of STAT1, STAT5, and STAT6, IL‐6 induced increased phosphorylation of STAT3 in Tregs of PSC‐patients (*p* = .0434). Finally, STAT3 activation in Tregs correlated with leukocyte counts.

**Conclusions:**

In PSC, we observed enhanced STAT3 responsiveness of CD4^+^ Tregs together with reduced CD39 expression probably reflecting inflammatory activity of the disease.

## Lay summary

We demonstrated that PSC is associated with reduced Treg frequencies and low CD39 expression in combination with increased levels of inflammatory cytokines such as IL‐6. Moreover, IL‐6 exposure leads to enhanced STAT3 activation in Tregs, which further contributes to reduced inhibitory function and increased inflammatory activity in PSC.

## Graphical abstract

We demonstrated that primary sclerosing cholangitis (PSC) is associated with reduced Treg frequencies and low CD39 expression in combination with increased levels of inflammatory cytokines such as interleukin (IL)‐6. Moreover, IL‐6 exposure leads to enhanced STAT3 activation in Tregs, which further contributes to reduced inhibitory function and increased inflammatory activity in PSC.

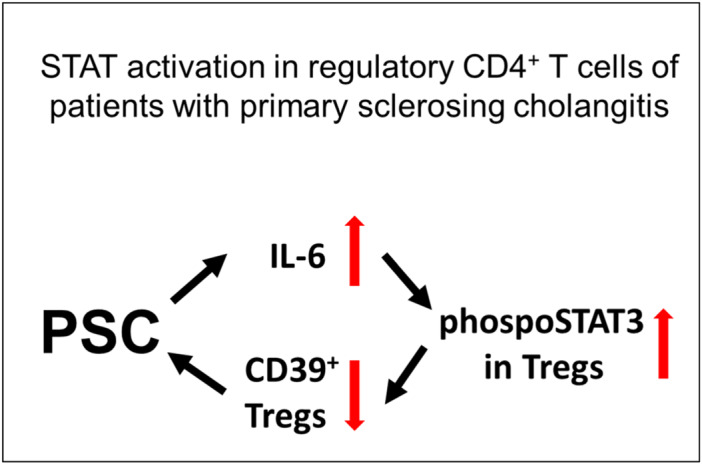

## 1 INTRODUCTION

Primary sclerosing cholangitis (PSC) is an autoimmune liver disease characterized by inflammation and fibrosis of the intra‐ and extrahepatic bile ducts.^1^, ^2^, ^3^ PSC often progresses to end‐stage liver disease, cirrhosis, and hepatobiliary malignancy^4^, ^5^ and is frequently associated with inflammatory bowel disease (IBD). To date there is no curative treatment that can slow progression of PSC.^6^, ^7^ Pathogenic mechanisms underlying PSC are still not fully understood but are likely to be multifactorial. Various findings suggest that genetic susceptibility and impaired immunoregulatory networks contribute to inflammation in PSC.^8^, ^9^

Regulatory CD4^+^ T cells (Tregs) are critical for the regulation of immune responses because they exert several anti‐inflammatory properties.^10^ Tregs inhibit maturation and function of antigen presenting cells and effector cells by secreting inhibitory cytokines or disrupt metabolic pathways (e.g., via activity of ATP/ADP ectonucleotidase CD39).^11^ Although in PSC higher frequencies of liver infiltrating Tregs^12^ and elevated numbers of circulating Tregs were initially reported,^13^ it is now assumed that patients with PSC exhibit a reduced number and impaired function of Tregs in both liver and blood.^14^, ^15^, ^16^ Recently, it has been reported that intrahepatic naive CD4^+^ T cells are prone to develop a pro‐inflammatory T helper type 17 (TH17) rather than a Foxp3^+^ Treg polarization state in patients with PSC.^17^

The Janus kinase (JAK)‐signal transduction and activation of transcription (STAT) pathways play a central role in the control of the immune system and modulate differentiation and activation of T helper cells and Tregs^18^: STAT1/STAT4 are key factors for TH1 cell polarization,^19^ STAT3 is required for the induction of TH17 cells,^20^ STAT5 promotes Treg differentiation by regulating Foxp3 expression,^21^ and STAT6 is an important regulator of TH2 signaling.^22^ Of note, interaction between STAT3 and STAT5 controls generation and activity of Tregs.^23^ Importantly, differentiation of naïve T cells into Tregs is impaired in vitro, when STAT3 is activated by IL‐6.^24^, ^25^ Furthermore, STAT3 activation in Tregs inhibits their suppressive function in metastatic melanoma^26^ and psoriasis.^27^, ^28^, ^29^ Recently, we demonstrated that IL‐6 triggers phosphorylation of STAT3 in TH1 and TH17 cells leading to enhanced production of IFN‐gamma and IL‐17A in PSC,^30^ while the role of differential activation of JAK‐STAT pathways has not yet been studied in Tregs from PSC‐patients.

Here, we analyzed frequencies and expression of CD39 in Foxp3^+^CD25^+^CD127^low^CD4^+^ Tregs in blood and studied activation of STAT1, 3, 5, and 6 proteins in response to recombinant cytokines. Findings were compared to serum cytokine levels and clinical data.

## 2 MATERIAL AND METHODS

### 2.1 Patients and controls

Fifty‐one patients with PSC were enrolled in this study. PSC diagnosis was based on the EASL clinical practice guidelines on sclerosing cholangitis.^31^ All patients with PSC had been monitored at our outpatient department at 3–4 monthly intervals. None of them had current bacterial cholangitis or biliary stent drainage. Patients with PSC were recruited during a routine visit at an average of 5.5 years after their first diagnosis of PSC. Concomitant IBD was diagnosed based on clinical criteria, and IBD phenotypes were classified as ulcerative colitis, Crohn's disease or indeterminate colitis.^2^, ^32^, ^33^, ^34^ Results were compared to healthy controls (*n* = 36) and disease control patients with chronic replicative hepatitis C (*n* = 10; median viral load 2,230,146; Abbott RealTime TaqMan HCV). All patients were recruited from the Department of Internal Medicine I at the University Hospital of Bonn and healthy controls from the Bonn University blood banking service, respectively.

Our study protocol followed the ethical guidelines of the Helsinki Declaration, and all participants had given their written informed consent as approved by the local ethics committee of the University Bonn (references: #003/2020 (PSC); #067/10 (chronic hepatitis C)). Due to the limited availability of study samples, we could not perform all tests in every individual. At recruitment, 40 mL of heparinized and 10 mL of clotted blood were collected simultaneously from each subject.

### 2.2 Reagents/antibodies

Foxp3/Transcription Factor Staining Buffer Set, True‐Phos™ Perm Buffer, Cell Staining Buffer, and recombinant human cytokines for in vitro stimulation (IFN‐gamma, interleukin (IL)‐6, IL‐2, IL‐4; all carrier‐free) were purchased from Biolegend.

The following antibodies were used to analyse Tregs via flow cytometry: anti‐CD4 (clone OKT4; APC‐Cy7‐labeled), anti‐CD3 (clone HIT3a; PE‐Cy7) anti‐CD25 (clone M‐A251; PerCP‐labeled), anti‐CD127 (clone A019D5; BV‐421‐labeled), anti‐Foxp3 (clone 206D; AlexaFluor647‐labeled), anti‐CD39 (clone A1; FITC‐labeled), and isotype controls (all from Biolegend). STAT activation was studied using commercially available PE‐labeled antibodies against phospho‐STAT1 (clone A17012A.Rec), phospho‐STAT3 (clone 13A3‐1), phospho‐STAT5 (clone A17016B.Rec), and phospho‐STAT6 (clone A15137E) (all Biolegend).

### 2.3 Cell preparations from peripheral blood

Peripheral blood mononuclear cells (PBMCs) were isolated by Ficoll‐Paque density gradient centrifugation (PAA Laboratories, Cölbe, Germany) from heparinized blood and cryopreserved in liquid nitrogen until analysis.

### 2.4 Measurement of cytokines in serum

IFN‐gamma, IL‐6, IL‐2, and IL‐4 were analyzed in serum samples from tubes with clotted venous blood. Sera from PSC‐patients and healthy controls were stored at −80°C until use. Cytokine concentrations were measured via a LEGENDplex™ bead‐based immunoassay using various anti‐human capture beads (IFN‐gamma, IL‐6, IL‐4, and IL‐2; Biolegend). Samples were analyzed on a FACSCanto II (BD Biosciences, Heidelberg, Germany) and cytokine levels were determined using the LEGENDplex™ cloud‐based software. Due to the limited availability of serum samples, we could not perform measurement of cytokines in every individual.

### 2.5 Analysis of phospho‐STATs in Tregs

In PBMCs we analyzed activation of STATs in fixed, permeabilized Foxp3^+^CD25^+^CD127^low^CD4^+^ Tregs by multiparameter phospho‐flow cytometry using the established protocol of Li and Park (https://www.biolegend.com/en-us/bio-bits/phospho-staining-and-intracellular-flow-cytometry).^35^

**Figure 1:**
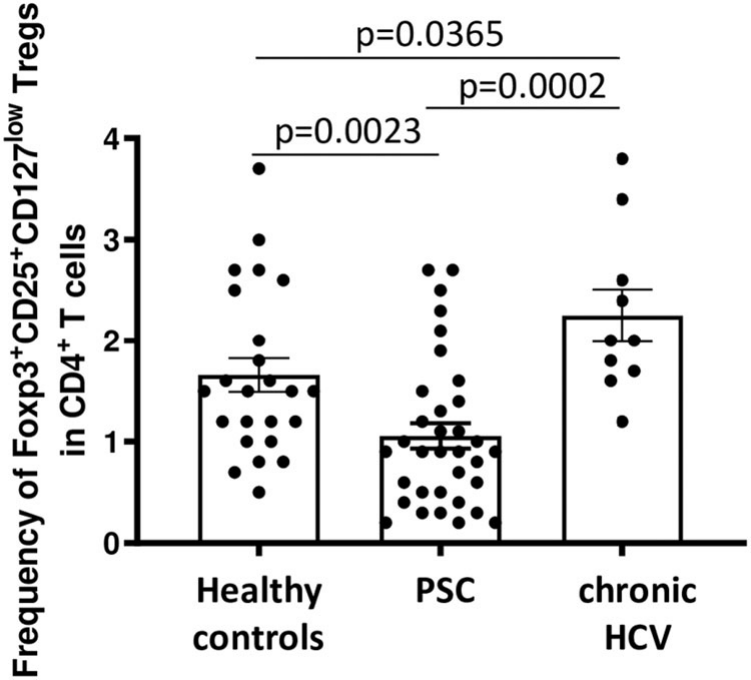
Frequencies of peripheral Foxp3^+^CD25^+^CD127^low^CD4^+^ Tregs in PSC, chronic replicative hepatitis C, and healthy controls. This figure demonstrates that frequencies of Foxp3^+^CD25^+^CD127^low^CD4^+^ Tregs were significantly reduced in patients with PSC (*n* = 34) compared to healthy controls (*n* = 24) and Tregs from patients with chronic hepatitis C (*n* = 10). Boxes and whiskers indicate mean and SEM, respectively. *p*‐Values refer to significances obtained by unpaired nonparametric Mann–Whitney test for the differences marked by bars. PSC, primary sclerosing cholangitis; SEM, standard error of mean.

Briefly, phospho‐STATs 1/3/5/6 were studied in PBMCs at baseline and after in vitro stimulation (37°C, 5% CO_2_) with recombinant cytokines (IFN‐gamma to induce phospho‐STAT1, IL‐6 to induce phospho‐STAT3, IL‐2 to induce phospho‐STAT5, IL‐4 to induce phospho‐STAT6) (50 ng/mL each). After 15 min, cells were washed with PBS and dead/viable cell discriminated by Zombie Aqua™ staining (10 min; BioLegend). Then, cells were stained with anti‐CD3, anti‐CD4, anti‐CD25, and anti‐CD127, respectively. Next, cells were incubated with Foxp3 Fixation/Permeabilization solution for 30 min on ice. After adding Fc blocking solution, cells were stained with anti‐Foxp3 for 30 min. Then, cells were washed again with Foxp3 Permeabilization Buffer (1X), resuspended in True‐Phos™ Perm Buffer and incubated overnight at −20°C in the freezer. Next day, cells were thawed, washed with Cell Staining Buffer and stained with phospho‐STAT antibodies in Cell Staining Buffer. Following 30 min incubation in the dark, cells were washed, resuspended in Cell Staining Buffer, and measured on a FACSCanto II (BD Biosciences). Using the FlowJo V10.8 software (TreeStar Inc.), we analyzed frequencies of Tregs via gating strategy using common Treg‐specific markers including Foxp3, CD25, CD127 (low), and CD39. In addition, expression of phospho‐STAT proteins in Tregs was determined at baseline and after in vitro stimulation. Our gating is shown in Supporting Information S1: Figure 1. FACS antibodies had been titrated in preceding experiments to find optimal dilutions. Isotype controls and fluorescence minus one (FMO) were done in all experiments. Due to the limited availability of PBMCs, we could not perform analysis of phospho‐STATs in every individual.

### 2.6 Statistical analysis

Data were analyzed using the IBM SPSS Statistics software (version 29; IBM) and GraphPad Prism (version 9.0; GraphPad Prism). Datasets were first tested for normality and then analyzed by the unpaired non‐parametric Mann–Whitney test, Wilcoxon matched‐pairs signed‐rank test and Student's *t*‐test as appropriate. Correlations between experimental results and clinical data were compared by Spearman rank correlation analyses. *p* < .05 was regarded to indicate statistical significance.

## 3 RESULTS

Our study included 30 male and 21 female patients with PSC. None of them had small duct PSC nor features of a PSC/AIH variant syndrome. Patients did not have acute bacterial cholangitis or biliary stent drainage at time of recruitment. Nine patients (18%) had liver cirrhosis as determined by combined methods such as transient elastography, MRI (magnetic resonance imaging) and ultrasound. Seventeen patients (33%) had high‐grade strictures. Thirty‐seven (73%) patients had concomitant chronic IBD (31 ulcerative colitis, 5 Crohn's disease, 1 indeterminate colitis) which was in remission in all patients as demonstrated by colonoscopy. Oral mesalazine (1.0–4.5 g/day) was the only given treatment needed in 27 (52%) patients. Forty‐four patients (86%) were on treatment with ursodeoxycholic acid (15 ± 5 mg/kg body weight). Our PSC‐cohort mainly consisted of patients with early disease as indicated by a median Amsterdam‐Oxford score of 1.4, median Meld score of 6, and median liver stiffness of 7.1 kPa. Clinical features and demographic data of our patient cohorts and healthy blood donors are summarized in Table 1.

**Table 1:** Characteristics of the study cohorts.

	Healthy controls	PSC	Chronic HCV
Total number of individuals	36	51	10
Age (years)	41 (20–79)	42 (20–67)	53 (25–63)
Sex (male/female)	23/13	30/21	5/5
Clincal parameters			
Amsterdam‐Oxford Score^§^	n.a.	1.40 (0.27–3.98)	n.a.
MELD Score	n.a.	6 (6–18)	6 (6–10)
Transient elastography (kPa)	n.d. [< 9.5]	7.1 (3.8–75.0)	8.2 (5.8–24.9)
AST (U/L)	n.d. [# < 50; <35]	33.0 (8.0–359.0)	45.5 (14.0–117.0)
ALT (U/L)	n.d. [# < 50; <35]	36.0 (7.0–771.0)	71.0 (21.0–172.0)
AP (U/L)	n.d. [# 40–130; 35–105]	141.0 (48.0–508.0)	54.0 (45.0–64.0)
Bilirubin (mg/dL)	n.d. [# < 1.4; <0.9]	0.66 (0.22–5.40)	0.46 (0.26–1.81)
CRP (mg/L)	n.d. [0–3]	1.77 (0.60–32.46)	n.d.
Leukocytes (g/L)	n.d. [3.9–10.2]	6.12 (2.83–12.46)	6.82 (5.04–13.14)
Platelets (Giga/L)	n.d. [150–370]	264 (45–497)	210 (136–300)

*Note*: (): Values are given as medians and ranges; []: Values indicate reference ranges (male; female); #: reference range in male, female patients; §: Amsterdam‐Oxford Score:https://sorted.co/psc-calculator/.

Abbreviations: ALT, alanine aminotransferase; AP, alcaline phosphatase; AST, aspartate aminotransferase; CRP, C‐reactive protein; n.a., not applicable; n.d., not done.

First, we analyzed frequencies of Foxp3^+^CD25^+^CD127^low^CD4^+^ Tregs in PBMCs of patients with PSC and the control groups. We found reduced frequencies of peripheral Foxp3^+^CD25^+^CD127^low^CD4^+^ Tregs in patients with PSC as compared to healthy controls, whereas frequencies of Tregs were markedly enhanced in patients with chronic hepatitis C (Figure 1). The role of increased frequencies of CD4^+^ Tregs has been extensively studied in chronic hepatitis C,^36^, ^37^, ^38^, ^39^, ^40^ whereas reduced CD4^+^ Tregs in PSC is a relatively new finding.^14^, ^15^, ^16^ Thus, we focussed our further studies on Tregs from patients with PSC.

**Figure 2:**
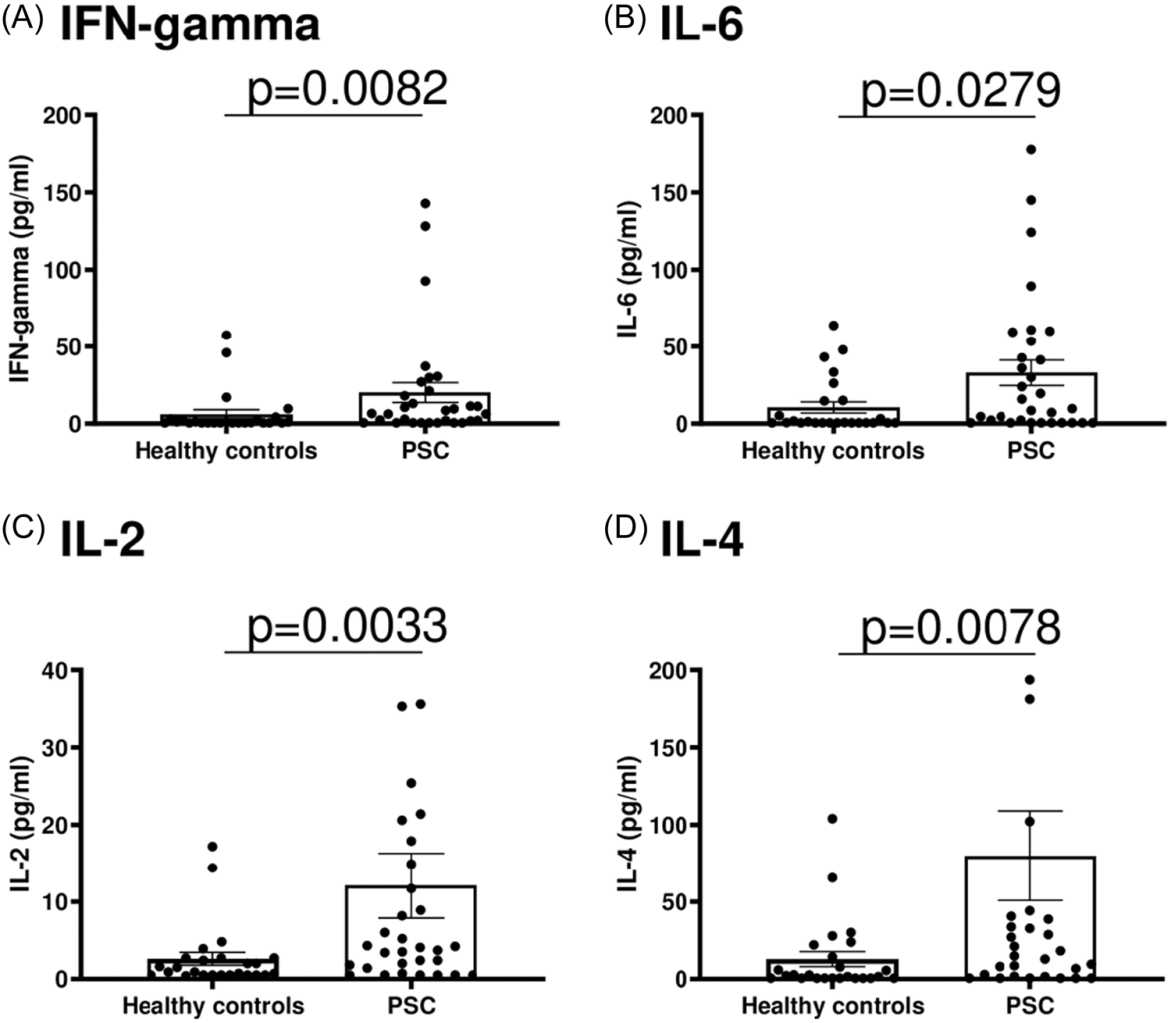
Cytokine levels in serum. These figures show cytokine serum levels in PSC‐patients (*n* = 31) compared to healthy controls (*n* = 25). Levels of IFN‐gamma (A), IL‐6 (B), IL‐2 (C), and IL‐4 (D) were significantly higher in PSC than in healthy controls. Boxes and whiskers indicate mean and SEM, respectively. *p*‐Values refer to significances obtained by unpaired nonparametric Mann–Whitney test for the differences marked by bars. IL, interleukin; PSC, primary sclerosing cholangitis; SEM, standard error of mean.

Since CD4^+^ Tregs control other immune cells and their cytokine production, we further analyzed levels of pro‐inflammatory/regulatory cytokines in the serum of patients with PSC and healthy controls. Our data revealed higher serum levels of IFN‐gamma, IL‐6, IL‐2, and IL‐4 in PSC than in healthy controls (Figure 2).

**Figure 3:**
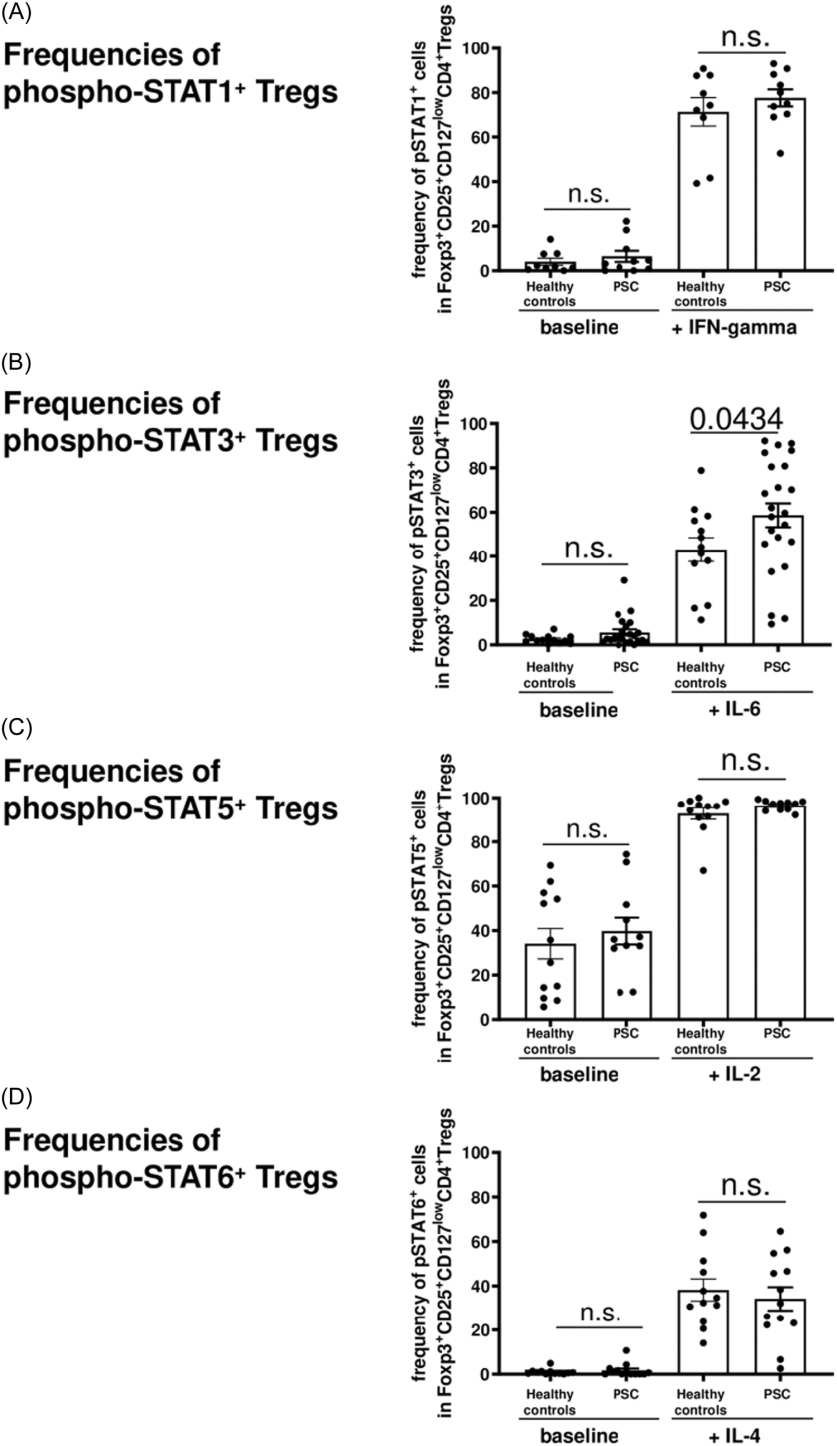
Expression of phospho‐STAT1, 3, 5, and 6 in Foxp3^**+**^CD25^+^CD127^low^CD4^+^ Tregs. Figures illustrate that – in contrast to phospho‐STAT1 (A), phospho‐STAT5 (C), and phospho‐STAT6 (D) – in vitro stimulation with recombinant cytokines resulted in enhanced frequencies of phospho‐STAT3‐positive Foxp3^+^CD25^+^CD127^low^CD4^+^ Tregs in PSC‐patients (*n* = 23) compared to healthy controls (*n* = 13) (B). Boxes and whiskers indicate mean and SEM, respectively. *p*‐Values refer to significances obtained by unpaired nonparametric Mann–Whitney test for the differences marked by bars. PSC, primary sclerosing cholangitis; SEM, standard error of mean.

Activation of Tregs is regulated via STAT proteins in response to certain cytokines: IFN‐gamma activates phosphorylation of STAT1, IL‐6 of STAT3, IL‐2 of STAT5, and IL‐4 of STAT6, respectively. To investigate potential effects of enhanced cytokines in PSC on the activation of STAT proteins in Tregs, we compared expression of phospho‐STAT1, 3, 5, and 6 in peripheral Foxp3^+^CD25^+^CD127^low^CD4^+^ Tregs between patients with PSC and healthy controls after in vitro stimulation with recombinant IFN‐gamma, IL‐6, IL‐2, and IL‐4, respectively. Due to their high turnover, baseline levels of STAT proteins were rather low and did not reach any differences between PSC and healthy controls. In vitro stimulation of PBMCs with cytokines induced phosphorylation of all corresponding STAT proteins (Figure 3). Of note, IL‐6‐induced activation of STAT3 in Foxp3^+^CD25^+^CD127^low^CD4^+^ Tregs was about 25% greater in patients with PSC than in the healthy controls (*p* < .05; Figure 3B). No such differences were detected for IFN‐gamma‐activated phospho‐STAT1^+^ Tregs (Figure 3A), IL‐2‐activated phospho‐STAT5^+^ Tregs (Figure 3C) or IL‐4‐activated phospho‐STAT6^+^ Tregs (Figure 3D). Enhanced phospho‐STAT3 expression in Tregs of patients with PSC could not be explained by increased IL‐6‐induced proliferation of Tregs, because frequencies of Tregs did not differ between baseline and IL‐6‐stimulation (Supporting Information S2: Figure 2).

**Figure 4:**
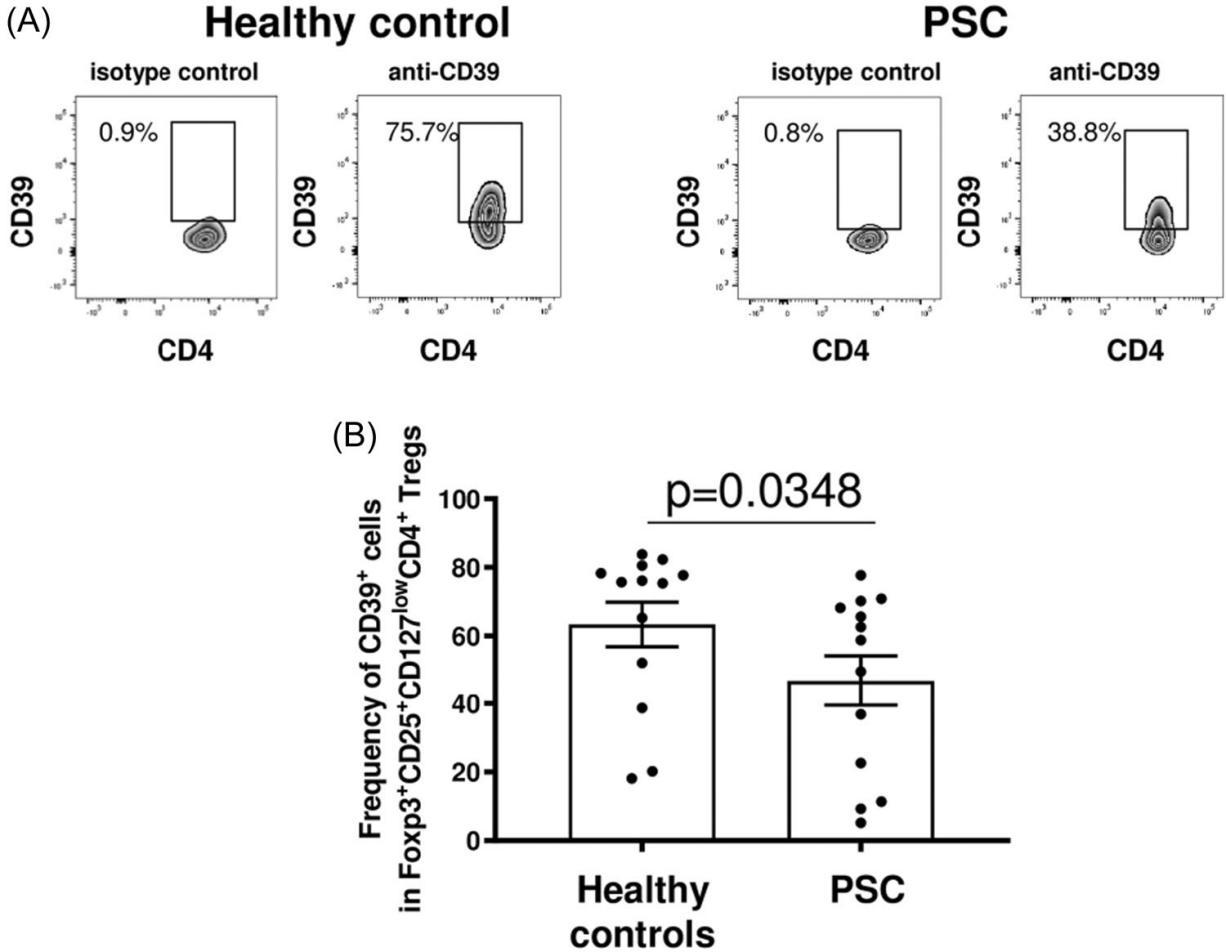
Expression of CD39 in Foxp3^+^CD25^+^CD127^low^CD4^+^ Tregs from patients with PSC and healthy controls. These figures demonstrate that frequencies of CD39‐positive cells in Foxp3^+^CD25^+^CD127^low^CD4^+^ Tregs were reduced in PSC (*n* = 13) compared to healthy controls (*n* = 13). Figure (A) illustrates representative flow cytometric zebra plots of CD39‐positive cells in Foxp3^+^CD25^+^CD127^low^CD4^+^ Tregs. Figure (B) shows the summary statistics in healthy controls and PSC patients. Boxes and whiskers indicate mean and SEM, respectively. *p*‐Values refer to significances obtained by unpaired non‐parametric Mann–Whitney test for the differences marked by bars. PSC, primary sclerosing cholangitis; SEM, standard error of mean.

A relationship between phosphorylation of STAT3 and expression of ectonucleotidase CD39 in Tregs has previously been reported under inflammatory conditions.^41^ Thus, we analyzed frequencies of CD39‐positive Tregs in PSC and found significantly reduced frequencies of CD39‐expressing Tregs in the PSC patients compared to healthy controls (Figure 4).

It has been proposed that immune responsiveness differs between males and females probably reflecting higher frequencies of Tregs in males.^42^, ^43^, ^44^ To address this issue in PSC, we reanalyzed our findings separately in male and female patients but observed similar changes in either sex. However, the greatest differences to healthy controls were observed in male patients with PSC (Supporting Information S3: Figure 3). Furthermore, frequencies of Tregs and their CD39 expression, IL‐6 serum levels, and phospho‐STAT3^+^ Tregs were not related to the presence of IBD (Supporting Information S4: Figure 4).

**Figure 5:**
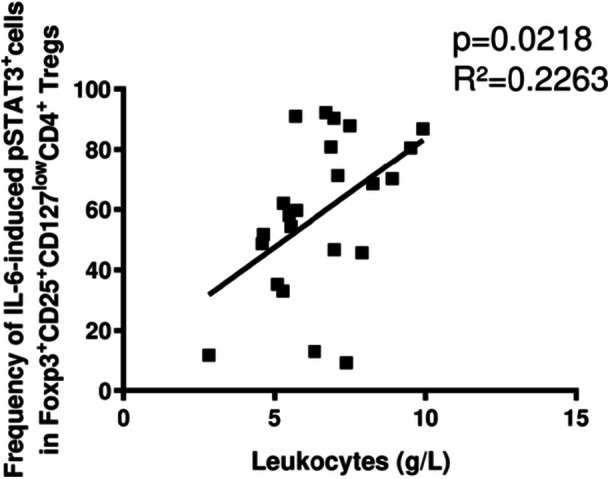
Relationship between phospho‐STAT3^+^Foxp3^+^CD25^+^CD127^low^CD4^+^ Tregs and leukocyte counts in PSC. This figure illustrates that frequencies of phospho‐STAT3‐positive Foxp3^+^CD25^+^CD127^low^CD4^+^ Tregs correlate with leukocyte counts (*n* = 23). *p*‐Value and the correlation coefficient refer to Spearman rank correlation analysis. PSC, primary sclerosing cholangitis.

Finally, we compared induction of phospho‐STAT3^+^ Tregs in PSC with clinical parameters and found that frequencies of phospho‐STAT3^+^ Tregs correlated with leukocyte counts (Figure 5).

## 4 DISCUSSION

Here, we report a detailed analysis of STAT activation in Tregs in PSC. First, we analyzed frequencies of Foxp3^+^CD25^+^CD127^low^CD4^+^ Tregs in peripheral blood and serum cytokine levels of patients with PSC and compared them to controls. In addition, we compared in vitro activation of various STAT proteins in Tregs upon cytokine stimulation and their CD39 expression between patients with PSC and healthy controls.

In PSC, overall frequencies of Foxp3^+^CD25^+^CD127^low^CD4^+^ Tregs were reduced, and serum levels of IFN‐gamma, IL‐6, IL‐2, and IL‐4 were markedly enhanced. These observations are in line with previous reports, which suggest that in PSC frequencies/functions of Tregs are reduced^14^, ^15^, ^16^ while levels of inflammatory cytokines are enriched.^45^ Importantly, we found that frequencies of Tregs were reduced in male patients with PSC in combination with high IL‐6 serum levels in line with the male predisposition for PSC.^2^, ^3^, ^6^

Tregs have been shown to be unstable in the presence of pro‐inflammatory cytokines and are regulated by the local milieu involving activation via JAK‐STAT pathways.^46^ Here, our data suggest a possible link between IL‐6 production and Treg activity because in PSC, we found increased frequencies of IL‐6‐induced phospho‐STAT3‐positive Tregs and reduced expression of ectonucleotidase CD39.

STAT3 is a key regulator in controlling autoimmunity^47^ which in combination with STAT1 and STAT5 also regulates functions of Tregs.^23^ Whereas STAT5 is a key positive regulator of Foxp3 expression, STAT3 has been shown to be an important inhibitor of Treg function.^48^ For instance, phospho‐STAT3 binds to a silencer element within the Foxp3 gene locus and is associated with a reduction in Smad3 binding.^49^ Importantly, STAT3 activation has been shown to impair differentiation of naïve T cells into Tregs^23^, ^25^, ^50^ and to promote instability of natural Tregs by limiting generation of induced Tregs.^24^ Furthermore, activation of STAT3 in Tregs was associated with decreased suppressive function in patients with metastatic melanoma.^26^ Even closer to the findings of our study in PSC, high exposure to IL‐6 in lesional tissue led to a dampened function of Tregs in patients with psoriasis.^27^, ^28^, ^29^ Furthermore, IL‐6 has been reported to be a suppressor of Foxp3 and other core signature genes of Tregs by transcriptional and posttranscriptional mechanisms.^51^

In this context, it was demonstrated that ectonucleotidase CD39, which catalyzes the hydrolysis of extracellular adenosine tri‐ and diphosphates into monophosphates,^11^ is downregulated in several systemic autoimmune diseases including IBD and autoimmune hepatitis.^52^ Furthermore, it has been shown that CD39 modifies biliary injury and fibrosis in a mouse model of PSC.^53^ Here, we confirmed markedly reduced frequencies of CD39‐positive Tregs in the blood from patients with PSC. Of note, Gu et al. reported that in the presence of IL‐6, CD39^high^ Tregs maintained stable Foxp3 expression, whereas CD39^low^ Tregs lost their inhibitory function and trans‐differentiated into TH1 and TH17 cells upon STAT3 activation.^41^ Thus, decreased CD39 expression in Tregs combined with their enhanced IL‐6‐induced STAT3 phosphorylation is in line with the concept of Treg trans‐differentiation into TH1/TH17 cells and may explain why also effector T cells in PSC show enhanced IL‐6‐dependent STAT3 activation.^30^

Although our data were obtained in peripheral blood and in vitro only, results may reflect clinically important findings because the correlation of phospho‐STAT3‐positive Tregs to leukocyte counts may indicate a relationship to inflammatory disease activity in PSC. However, this intriguing hypothesis still needs to be confirmed by further studies taking into account that an established surrogate marker of inflammatory activity in PSC is still missing.^54^

Furthermore, our observations may become important when autologous transfer of Tregs should become a novel strategy to address PSC^55^ since our data suggest to keep an eye on CD39 expression and STAT3 activation in adoptively transferred Tregs to optimize their therapeutic benefits considering the possibility that their regulatory functions might gradually deteriorate due to IL‐6 exposure in the local milieu.

## AUTHOR CONTRIBUTIONS

**Leona Dold**: Conceptualization; methodology; project administration; writing—original draft; writing—review & editing. **Sandra Kalthoff**: Conceptualization; methodology; writing—original draft; writing—review & editing. **Leonie Frank**: Formal analysis; funding acquisition; methodology; writing—review & editing. **Taotao Zhou**: Formal analysis; project administration; writing—review & editing. **Pia Esser**: Methodology; writing—review & editing. **Philipp Lutz**: Project administration; writing—review & editing. **Christian P Strassburg**: supervision; writing—review & editing. **Ulrich Spengler**: Conceptualization; formal analysis; methodology; writing—original draft. **Bettina Langhans**: Conceptualization; funding acquisition; methodology; project administration; writing—original draft; writing—review & editing.

## CONFLICT OF INTEREST STATEMENT

The authors declare no conflict of interest.

## Supporting information

Supporting information.

Supporting information.

Supporting information.

Supporting information.

## Data Availability

The data of this study are available upon request.
