# Supplementary figures and images for: STAT activation in regulatory CD4+ T cells of patients with primary sclerosing cholangitis
Source: Immun Inflamm Dis. 2024 Apr 12;12(4):e1248. doi: 10.1002/iid3.1248 (PMC11010953; doi:10.1002/iid3.1248)

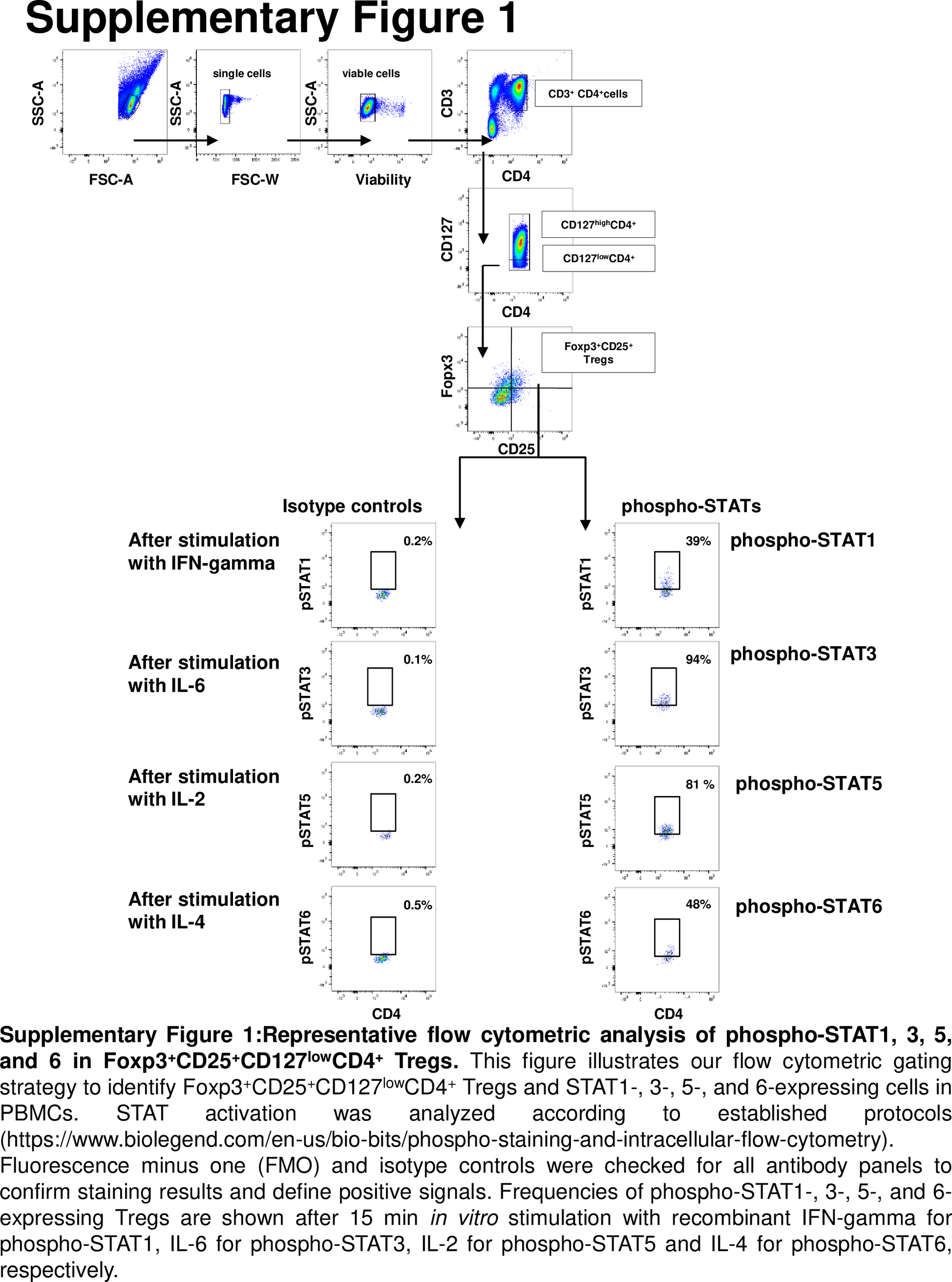

Supplement: Supplementary file 1 — Supporting information. [file IID3-12-e1248-s004.tif]

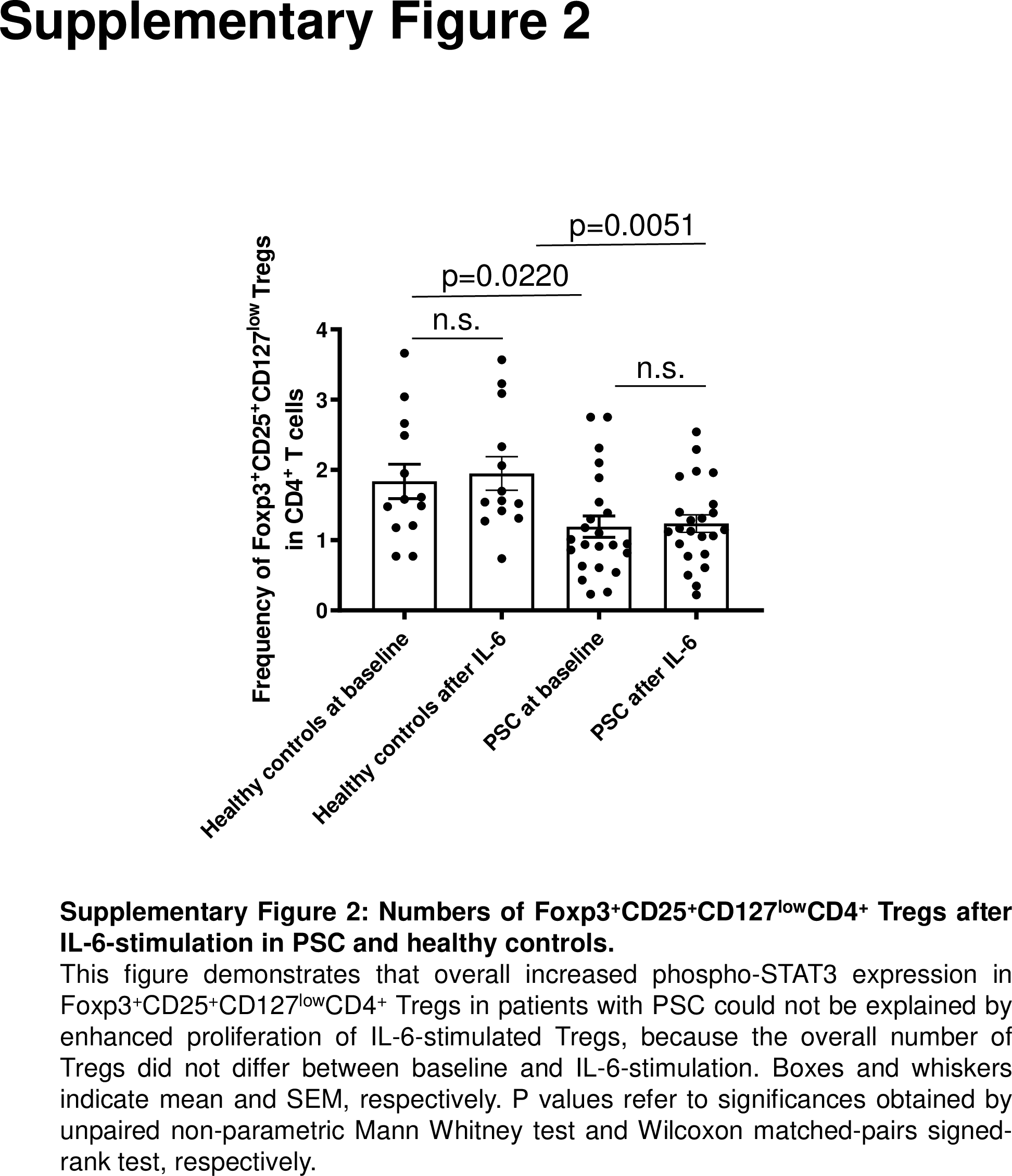

Supplement: Supplementary file 2 — Supporting information. [file IID3-12-e1248-s001.tif]

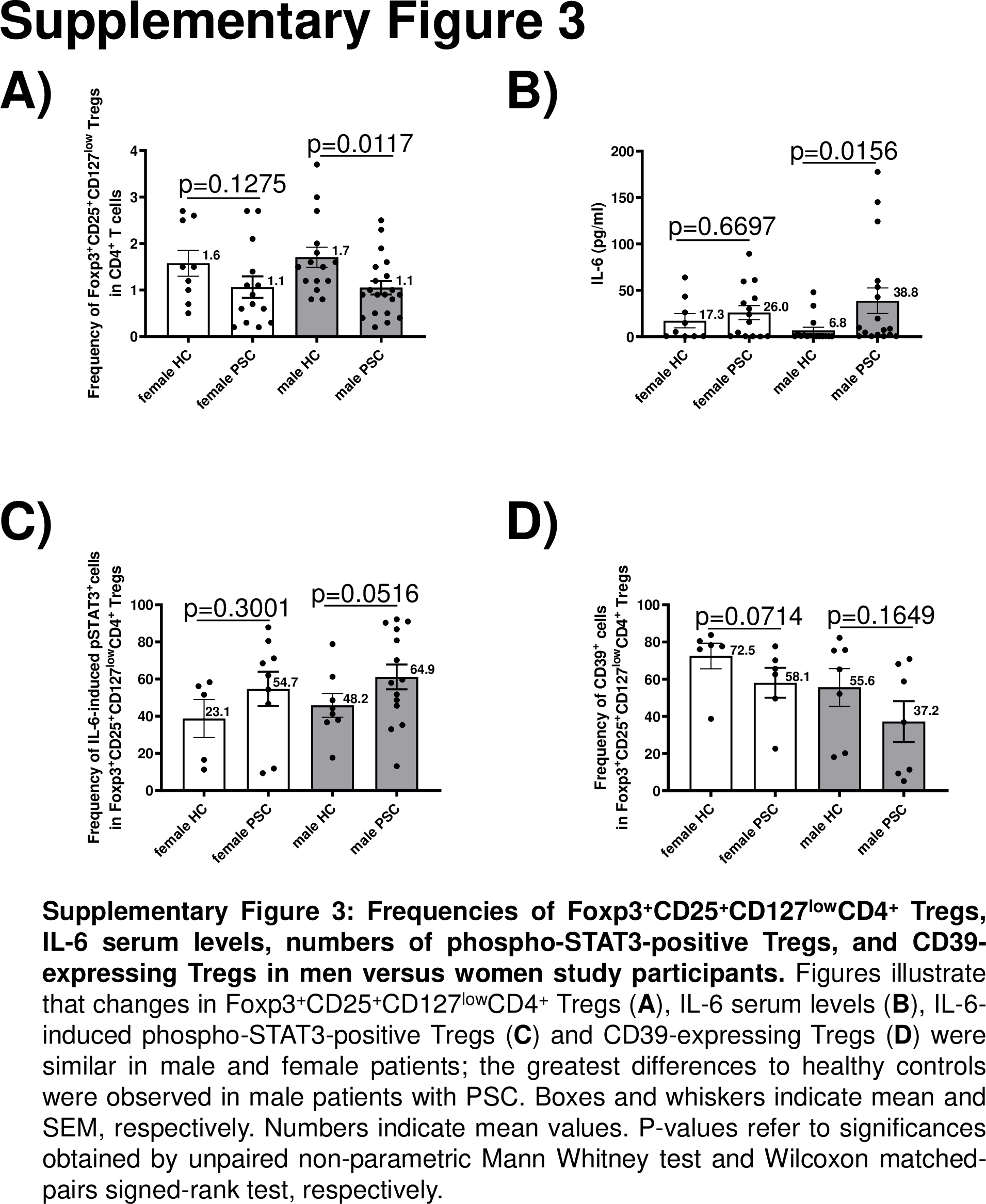

Supplement: Supplementary file 3 — Supporting information. [file IID3-12-e1248-s002.tif]

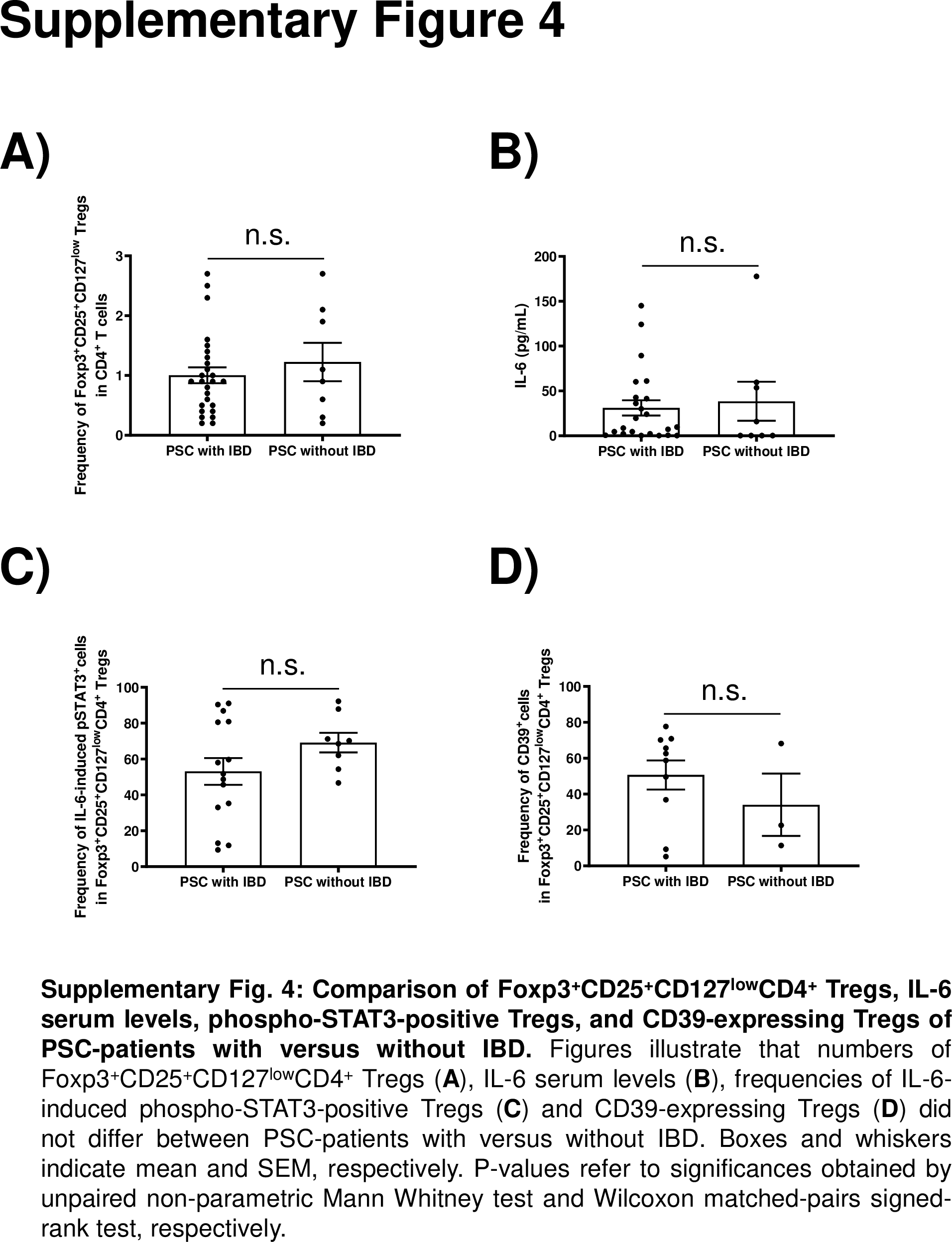

Supplement: Supplementary file 4 — Supporting information. [file IID3-12-e1248-s003.tif]
